# Supplementary material for: Biological sample donation and informed consent for neurobiobanking: Evidence from a community survey in Ghana and Nigeria
Source: PLoS One. 2022 Aug 11;17(8):e0267705. doi: 10.1371/journal.pone.0267705 (PMC9371301; doi:10.1371/journal.pone.0267705)
Supplement: S1 File — (PDF) [file pone.0267705.s001.pdf]

Study ID ____________________

African Neurobiobank for Precision Stroke Medicine - Ethical, Legal, and Social Implications (ELSI) Project

DRAFT QUESTIONNAIRE SIREN STROKE CASES, SIREN CONTROLS AND LAY PERSONS

Dear Respondent,

Good day. We are conducting a survey to explore your knowledge, attitude and perceptions, about ethical, legal and social issues related to the use of blood and stored blood fractions, brain images (CT/MRI) and brain donation in the context of stroke genomic research.

We invited you to respond to a couple of questions because we believe that you have one important thing or the other to share with us on issues related to ethical, legal and social issues relating to stroke biobanking in the African context.

Whatever we learn from today's discussion will help us develop an intervention program to address the ELSI issues related to stroke genomic and biobanking research in Sub Saharan Africa.

We will not write your name or house address on the questionnaire. Some of the questions may touch on very personal issues. Your true opinions on these issues will be greatly appreciated. Please, kindly answer all questions sincerely. You have a right to decline to participate in the study without any consequence.

Thank you.

Willingness to participate Yes ☐ No ☐

Please tick the category the participant belongs to.

- ☐ SIREN Enrolled Stroke cases
- ☐ SIREN Enrolled Stroke Free Controls
- ☐ Non SIREN Stroke Free Lay Persons

Mode of questionnaire administration (PIs tick one option as applicable)

- ☐ Interviewer Administered
- ☐ Self-Administered

Interviewers Name/Initials ____

SECTION A: Socio Demographic Characteristics

1. Age in years (as at last birthday) ____________________ 2. Gender Male ☐ Female ☐

3. Type of Domicile: Rural ☐ Semi-urban ☐ Urban ☐

4. Religion: Christianity ☐ Islam ☐ Traditional ☐ Others (specify) ____________

5. Native Language: Yoruba ☐ Igbo ☐ Hausa ☐ Aka ☐ Ga/Adangbe ☐ Ewe ☐ Others (specify)____________

6. Ethnic group: Yoruba ☐ Igbo ☐ Hausa ☐ Aka ☐ Ga/Adangbe ☐ Ewe ☐ Others (specify)____________

7. Country of birth: Nigeria ☐ Ghana ☐ Others (specify)____________

8. Average Total Monthly Household Income in USD: 0 -100 ☐ 101 -250 ☐ 251 -500 ☐ 501 - 1500 ☐ 1501 - 3000 ☐ >3000 ☐

9. Marital Status: Single ☐ Married ☐ Widowed ☐ Separated ☐ Divorced ☐

10. Living Situation: Lives alone ☐ Lives with spouse and children ☐ Lives in a nursing home ☐ Live with spouse ☐ Lives with extended family ☐ Lives with children ☐ Others (specify)____________

11. Primary Occupation: ____________________

11b. Classification: Highly skilled academic profession ☐ Home maker ☐ Skilled worker ☐ Retired ☐ Manual work ☐ Unemployed(able to work) ☐ Student ☐ Non-paid worker ☐ Semi-skilled worker ☐ Unemployed (unable to work) ☐

11c. If retired, Primary Occupation before retirement: ____________________

12. Educational qualification: None ☐ Informal education ☐ Arabic School ☐ Primary ☐ Junior secondary ☐ Senior secondary ☐ Tertiary ☐ Postgraduate ☐

SECTION B: Awareness and knowledge of blood sample donation and bio-banking. *Tick (√) the option appropriate to you)*

13. Have you ever heard of the following terms?

a. Blood sample donation for medical research? Yes ☐ No ☐

b. Blood sample storage (bio-banking) for research purpose? Yes ☐ No ☐

If No to both questions a and b, please go to question 18.

14. If Yes, what do you know about blood sample donation for research? ____________________

____

____

15. If Yes, what do you know about blood sample storage (bio-banking) for research purpose? ____________________

____

____

16. If your answer to question13a above is Yes, from which of the following sources did you hear about blood sample donation? (***You can choose more than one option).***

Hospital ☐ Training program ☐ Friend ☐ Colleague ☐ Newspaper/magazine ☐ Internet/Online resourcess ☐

Seminar/conference/workshop ☐ TV ☐ Radio ☐ Community/public outreach/enagement Initiative ☐ Family ☐ Others(Specify)____________

17. If your answer to question13a above is Yes, from which of the following sources did you hear about blood sample storage for research purpose? (***You can choose more than one option).***

Hospital ☐ Training program ☐ Friend ☐ Colleague ☐ Newspaper/magazine ☐ Internet/Online resourcess ☐

Seminar/conference/workshop ☐ TV ☐ Radio ☐ Community/public outreach/enagement Initiative ☐ Family ☐ Others(Specify)____________

18. Which of the following biological samples can be stored in a biobank? (***You can choose more than one option).***

Blood and related blood fractions ☐ Urine ☐ Cerebrospinal fluid ☐ Brain tissues ☐ Others(Specify)________________

19. Are you aware of any guidelines/regulation that has to do with use of blood and its storage for genomic research purpose? Yes ☐ No ☐

20. If Yes, do you have a copy of any guidelines/regulation that has to do with use of blood and its storage for genomic research purpose? Yes ☐ No ☐

SECTION C: Genetic Knowledge (*Please Tick* (√) *the option appropriate to you*)

SN	Statements	Yes	No	Don't Know
21	One can see a gene with the naked eye.			
22.	A gene is a disease.			
23.	A gene is a molecule that controls hereditary characteristics			
24.	Genes are inside cells			
25.	A gene is a piece of DNA			
26.	A gene is a cell			
27.	A gene is a part of a chromosome			
28.	Different body parts include different genes			
29.	Genes are bigger than chromosome			
30.	The genotype is not susceptible to human intervention			
31.	It has been estimated that a person has 22,000 genes			
32.	Healthy parents can have a child with a hereditary disease			
33.	The onset of certain diseases are due to genes, environment and lifestyle			
34.	The carrier of a disease gene may be completely healthy			
35.	All serious disease are hereditary			
36.	The child of a disease gene carrier ia always also a carrier of the same disease gene			

SECTION D: Awareness of Brain Donation for Research (*Please Tick* (√) *the option appropriate to you*)

37. Have you ever heard of brain donation for research purpose? Yes ☐ No ☐ ***(If answer is No, please go to question 41)***

38. If Yes, what did you hear about brain sample donation for research? ________________

39. If your answer to question 38 above is yes, through which of the following sources did you hear about brain sample for research purpose? (***You can choose more than one option).***

Hospital ☐ Training program ☐ Friend ☐ Colleague ☐ Newspaper/magazine ☐ Internet/Online resourcess ☐

Seminar/conference/workshop ☐ TV ☐ Radio ☐ Community/public outreach/enagement Initiative ☐ Family ☐ Others(Specify)________

40. Do you know anyone who donated brain for research purposes before? Yes ☐ No ☐

41. Have you ever heard of the concept of collecting and storing brain for research purposes? Yes ☐ No ☐ ***(If answer is No, please go to Q.44)***

42. If answer to question 41 above is Yes, through which of the following sources did you hear about banking/storing of brain for research (***You can choose more than one option).***

Hospital ☐ Training program ☐ Friend ☐ Colleague ☐ Newspaper/magazine ☐ Internet/Online resources ☐

Seminar/conference/workshop ☐ TV ☐ Radio ☐ Community/public outreach/enagement Initiative ☐ Family ☐ Others(Specify)________

43. Are you aware of any guidelines/regulation that has to do with use of brain and its storage for research purpose? Yes ☐ No ☐

44. Would you be willing to donate your brain sample for research purposes after death? Yes ☐ No ☐

44a. If Yes, please state your reasons **(you can choose more than one option)**

I like the idea that it will help advance medicine ☐

I like the idea that it will help prevent future disease ☐

I have no need of my brain again after death ☐

I would be happy knowing that I can save someone's life ☐

I like the idea that it can help generation to come ☐

Others(Specify)________________

44b. If No, please state your reasons **(you can choose more than one option)**

I want to go back to God complete ☐
my religion does not allow that ☐
if I donate my brain, people will think I have joined occultic association ☐
I am not knowledgeable of the purpose ☐
I don't trust Africans ☐
I don't trust medical system/medical researchers ☐
it's like someone is destroying the work of God ☐
I don't just want to ☐

Others(Specify)____

45. People in Africa would be willing to donate brain samples for research purposes? Agree ☐ Disagree ☐

45a. If you **Agree**, please state your reasons (allow respondent to give as many reasons as possible) ____________________

____

____

45b. If you **Disagree**, please state your reasons (allow respondent to give as many reasons as possible)

Most people are not knowledgeable about it importance ☐
Nobody will agree to buried with incomplete body parts ☐
It is not commonly done in our environment ☐
Others (Specify)________________________________

____

____

46. What kind of actions should be taken in order to promote willingness to donate brain samples for research? (***You can choose more than one option).***

Media publicity ☐ Education ☐ Legislation ☐ Involving religious and community leaders ☐ Education of people on social media ☐

Others (Specify) ________________________

SECTION E: Awareness of Stroke Genomic Research. (*Please Tick* (√) *the option appropriate to you*)

47. Have you ever heard of stroke genomic research? Yes ☐ No ☐ ***(If answer is No, please go to Section F)***

48. If your answer to question 47 above is **Yes**, through which of the following sources did you hear about stroke genomic research? (***You can choose more than one option).***

Hospital ☐ Training program ☐ Friend ☐ Colleague ☐ Newspaper/magazine ☐ Internet/Online resourcess ☐

Seminar/conference/workshop ☐ TV ☐ Radio ☐ Community/public outreach/enagement Initiative ☐ Family ☐ Others(Specify)__________

49. Have you ever donated blood for stroke genomic research before? Yes ☐ No ☐

50. Have you ever heard of the concept of collecting blood and storing the blood for stroke genomic research?

Yes ☐ No ☐ **If answer is No, please go to Section F.**

51. If answer to question 50 above is Yes, through which of the following sources did you hear about banking/storing of blood for stroke genomic research (***You can choose more than one option).***

Hospital ☐ Training program ☐ Friend ☐ Colleague ☐ Newspaper/magazine ☐ Internet/Online resourcess ☐

Seminar/conference/workshop ☐ TV ☐ Radio ☐ Community/public outreach/enagement Initiative ☐ Family ☐ Others(Specify)__________

52. Kindly tick the appropriate response for the following statements on blood collection:

SN	Statements	Yes	No	Don’t Know
	Research on blood collected can lead to advancement in medicine such as:			
1	Drug discovery?			
2.	Better management of illness			
3.	Diagnostic tools			

SECTION F: Awareness of Precision Medicine. (*Please Tick* (√) *the option appropriate to you*)

53. Have you ever heard of precision stroke medicine? Yes ☐ No ☐ ***(If answer is No, please go to Question 55)***

54a. If your answer to question 53 above is Yes, through which of the following sources did you hear about precision stroke medicine? (*You can choose more than one option).*

Hospital ☐ Training program ☐ Friend ☐ Colleague ☐ Newspaper/magazine ☐ Internet/Online resourcess ☐

Seminar/conference/workshop ☐ TV ☐ Radio ☐ Community/public outreach/enagement Initiative ☐ Family ☐ Others(Specify)____________

54b. If Yes, what is precision stroke medicine? ____

____

SECTION G: Awareness , Knowledge and Perception on Informed Consent Processes

55. Have you ever heard of informed consent before? Yes ☐ No ☐

56. What do you know about the informed consent process for genetic research?________________________

____

____

____

57. Types of informed consent preferred (Facilitator to please explain each type to the participants)

Broad ☐ Restricted ☐ Tiered ☐ Dynamic ☐

57a. If broad, provides reason(s) for your choice ____

____

57b. If restricted, provides reason(s) for your choice ____

____

57c. If tiered provides reason(s) for your choice ____

____

57d. If dynamic, provides reason(s) for your choice ____

____

58. Who are the persons to be involved before you can give informed consent? No one ☐ Spouse ☐ Children ☐ Parents ☐

Religious leaders e.g Pastors, Imam etc ☐ Others (Specify) ________________________

59. How should data collected from you be used in the incident of death?

Once I have given it, you can do anything good with it even after my death ☐

My children/family should be called to decides ☐

It should be discarded ☐

Others(Specify)______________________________

59a. Give reasons for your response ____

____

60. It is best to use generic informed consent for community? (Facilitator should please explain) Agree ☐ Disagree ☐

60a. If you **Agree**, please state your reasons (allow respondent to give as many reasons as possible) ________________________

____

____

60b. If you **Disagree,** please state your reasons (allow respondent to give as many reasons as possible) ________________________

____

____

For the following perception questions on Informed consent, please tick (√) the option appropriate to you

61	Perception	Agree	Not sure	Disagree
1	Broad informed consent should be used for genomic research			
2.	Consent forms should include a separate section relating to storage and future use of samples & data			
3.	It is personal choice to give blood for research.			
4.	Any blood sample collected from me must not be used for any other secondary use (subsequent use of stored samples for researches not indicated on the informed consent)			
5.	I will participate in genomic research if my community leader agrees			
6.	Donor must be contacted each time the sample is to be re-used.			
7.	I feel it's a criminal offence to make profit from sample collected from me.			

SECTION H: Willingness/Intention towards blood sample donation for research and biobanking *(please tick (√) the option appropriate to you)*

62. Would you be willing to give consent for blood sample donation for genetic research and saving for future use? Yes ☐ No ☐

63. Are there any particular reasons that can make you less likely to donate your blood sample for genetic research and banking for future use?
You can choose more than one answer.

This is against my religion ☐
I am not fat ☐
I don't trust medical system ☐
Blood is life and I don't want to reduce my lifespan. ☐
Never thought about it ☐
It may be used for rituals because I don't trust researchers ☐
My doctor warned me against donating blood . ☐
I fell terribly sick the last time I donated blood ☐

Others(Specify)____

64. What is/are could be your greatest motivation towards consenting to donating blood sample for genetic research?
(***You can choose more than one option***)

That the result would help prevent future diseases ☐
It will contribute to research activities ☐
It would give me a sense of responsibility ☐
It would give me a sense of pride ☐
I would be happy knowing that I can save someone's life ☐
I like the idea that it can help us know about our problems ☐
I like the idea that it can help generations to come ☐
Drugs could be developed that can impact positively on the society ☐

Others (Specify) ________________________________

65. If you were asked in a hospital to give consent for a family member blood sample donation for genetic research and banking for future use,

will you agree? Yes ☐ No ☐

65a. if yes, please give reasons for your answers ____

____

65b. If No, please give reasons for your answers ____

____

66. What kind of actions should be taken in order to promote willingness to give blood sample for research and banking for future use?
(***You can choose more than one option***)

Media publicity/campaign ☐ Public Education/Advocacy ☐ Legislation ☐ Involving religious and community leaders ☐

Education of people through social media ☐ Education by healthcare provider ☐ Others (Specify) __________________________

SECTION I Health conscious behaviours

66. What do you do purposely for your health (*Please choose as many as apply to you*)

Reduce alcohol consumption	☐	Avoid eating between meals	☐
Reduce amount of smoking	☐	Purposely eat less	☐
Exercise at least 30minutes or more once per week	☐	Refrain from oily foods	☐
Purposely move body once a week	☐	Reduce salt intake	☐
Try to eat fish	☐	Others ______________________________	

SECTION J: Data sharing, Use and Reuse of Stored Blood/Blood products, Brain Tissue samples and images

67. Blood/blood fractions taken from me can be shared with other researchers both locally and internationally.

Yes ☐ No ☐

67a. if yes, please give reasons for your answers ______________________________

____

67b. If No, please give reasons for your answers ______________________________

____

68. Brain tissue samples taken from me can be shared with other researchers both locally and internationally

Yes ☐ No ☐

if yes, please give reasons for your answers ______________________________

____

If No, please give reasons for your answers ______________________________

____

70. My brain images can be shared with other researchers both locally and internationally Yes ☐ No ☐

if yes, please give reasons for your answers ______________________________

____

If No, please give reasons for your answers ______________________________

____

SECTION K: Perception of biological sample donation for research and secondary use *(please tick ($\sqrt{}$) the option appropriate to you)*

71	Perception	Agree	Not sure	Disagree
i	Donating blood sample for research is good because any discovery can help to save lives.			
ii	Blood sample availability creates important opportunities for researchers to advance medical science and contribute to collective good.			
iii	Blood collected from me can be stored for future use since I have no need of it again.			
iv.	There is no need to contact the donor of the blood sample if the need arises to conduct future studies not previously planned.			
v	Every benefit/ profit/ money got through research on my blood sample should be shared with me.			
vi.	Blood sample collected from me can be taken to another country for further use.			
vii.	My religion does not allow blood sample donation for research.			
viii.	I will give blood sample if my pastor / imam agrees.			
ix.	It is wrong in my culture to give blood sample for research purposes			
x.	It is wrong in my culture to give blood sample for storage purposes			
xi.	I will like to receive the result of genetic testing done on my blood sample.			
xii.	Clients who desire to know their own genetic status should receive pre- and post-genetic counselling.			

71	Perception	Agree	Not sure	Disagree
xiii	Genetic discrimination in the context of their marriage, employment or insurance may increase if result of finding is returned to donor			
xiv.	Returning genetic test results from genetic studies, should be done using 'face-to-face' approach			
xv.	I would support my family members who shows willingness to donate blood sample for genomic research			
xvi.	I would support my family members who shows willingness to donate blood sample for storage			

Section L: Barriers and facilitators relating to blood donation (other biological samples) for research and bio-banking practices?

72. Have you ever donated blood or had other biological samples/reports been collected for research purpose before? Yes ☐ No ☐

73a. If Yes to question 68, kindly complete the table below:

SN	Biological Sample(s)	Organisation project	Date	Location	Purpose for which sample was collected/donated
a	Blood				
b	Urine				
c	Cerebrospinal fluid				
d	Brain Images(CT/MRI)				
e	Brain Tissues				
f	Others(specify)______ __________________				

73b. If your answer to question 68 is No, have you ever had the opportunity to donate blood or had your blood (or other biological samples/reports) collected as sample for research to which you declined? Yes ☐ No ☐

73bi. If Yes, kindly explain your reason(s) for declining to donate blood or have your blood (or other biological samples/reports) collected as sample for research ____

____

74. What factors will greatly encourage you to participate in blood donation and (or other biological samples/reports) collection for genomic research?

____

Section M: Perception relating to disclosure of individual genomic Research Findings?

75. Have you ever received genetic counselling before? Yes ☐ No ☐

75a. If yes, what were you counselled for? ____

76. Have you ever participated in genomic research before Yes ☐ No ☐ **if No go to Q.79**

77. If you had participated in a blood donation/other biological sample/reports collection activity, how was the result/research findings shared with you?

Please specify: Phone Call ☐ Email ☐ Letters ☐ Feedback by a health worker ☐ Feedback through a researcher ☐

Feedback through a clinician ☐ Others (please specify) ____

78. Did you have any issue relating to how your result/research finding was returned to you Yes ☐ No ☐

78a. If Yes, what were some of the issues you had with the means of research/result dissemination stated above? ______________________

____

79. If you are to participate in a blood donation/sample collection in genomic research, which way would you want your individual result or incidental findings to be relayed back to you? **You can choose more than one answer** Phone Call ☐ Email ☐ Letters ☐ Feedback by a health worker ☐

Feedback through a researcher ☐ Feedback through a clinician ☐ Others (please specify) ______________________

80. Will you prefer to have your individual genetic research/result findings collected by proxy or third party? Yes ☐ No ☐

SECTION N: Bio-rights

81. Do you think participants in researches should have control on how their biological specimens will be used? Yes ☐ No ☐ Don't know ☐

82. How much control should/can individuals have regarding how their biological specimens will be used in research?

None ☐ Little ☐ Much ☐ Total ☐

83. Has this interview influenced your willingness to give consent for blood sample donation for research and banking? Yes ☐ No ☐

83a. if yes, please give reasons for your answers ______________________________

____

83b. If No, please give reasons for your answers ______________________________

____
