# Supplementary material for: Biological sample donation and informed consent for neurobiobanking: Evidence from a community survey in Ghana and Nigeria
Source: PLoS One. 2022 Aug 11;17(8):e0267705. doi: 10.1371/journal.pone.0267705 (PMC9371301; doi:10.1371/journal.pone.0267705)
Supplement: Attachment — Submitted filename: reviewer response.docx [file pone.0267705.s003.docx]

16/02/2022

Dear Sir/Madam,

Please find attached our revised manuscript. All the authors have read and approved the revised version of the manuscript which comprises original, unpublished material that has not been considered for publication elsewhere.

We are grateful to the reviewers for their thoughtful comments. We have rewritten the paper in the light of their input and have responded to each comment below.

Detailed response to Reviewers’ comments:

| **Comments from reviewer 1** | | |  |
| --- | --- | --- | --- |
| 1 | Introduction  - The Introduction appears to be less well-developed than the Discussion and would benefit from elaboration. Specifically, it would help if the authors defined or described certain terms, such as “communal informed consent,” and provided more background on previous studies that were included as comparators in the Discussion.  - There is very little background information provided to explain the inclusion of certain survey questions or answer options | We thank the reviewer for this comment. The introduction has now been revised to include more background information on previous studies that were included in the discussion and also provided definition of communal informed consent.  The study questionnaire was developed by our strong multidisciplinary team with members who have expertise in mixed methods community-engaged research, bioethics, sociological, cultural and anthropological research and community leaders. Members of this team had previous relevant research experience in Africa, which informed the framing, review and updating of the willingness/unwillingness questions. | |
| 2 | Methods   - Line 147 – it is not clear why the stroke heritability results from this previous study are relevant in the sample size estimate. - Lines 171-176 – How were the answer options for the willingness/unwillingness questions determined? These appear to be unique to African communities and differ from what would typically be used in the U.S. or Europe. Explanation of data that informed these would be helpful for the general reader. - I appreciate the inclusion of all the survey questions in the manuscript but the current format leads to long, run-on sentences. It may be more useful to portray the survey outline as a table or figure. - It is not clear whether the participants received any brief education on certain topics to ensure they understood the survey questions, such as for the types of consent. This is implied at one point in the Discussion but this should be made clear in the Methods. - It would help to have more description of how participants were recruited. The authors state that they were selected during SIREN site community engagement activities but what brought them to be involved in these activities and what did these activities entail? Were these individuals patients, caregivers, other? How could this introduce bias? | We used knowledge of stroke heritability as a proxy for awareness about stroke genomics research, which was one of the goals of the ELSI project. We have included this statement in the paper form line 253-255.  Our strong multidisciplinary team developed the study questionnaire with members who have expertise in mixed methods community-engaged research, bioethics, sociological, cultural and anthropological research and community leaders. Members of this team had previous relevant research experience in Africa, which informed the framing, review and updating of the willingness/unwillingness questions.  We have now included a statement in the section on Study tool and Data Collection:  “The study questionnaire was developed by a multidisciplinary expert-working group, informed by the findings from a systematic review of the literature including previous studies (6,14–16)”  References  1. Akinyemi et al. Biobanking in a Challenging African Environment: Unique Experience from the SIREN Project. Biopreserv Biobank. 2018 Jun;16(3):217-232. doi: 10.1089/bio.2017.0113  2. Akinyemi et al. Brain banking in low and middle-income countries: Raison D'être for the Ibadan Brain Ageing, Dementia And Neurodegeneration (IBADAN) Brain Bank Project. Brain Res Bull. 2019 Feb;145:136-141. doi: 10.1016/j.brainresbull.2018.08.014.  3. Akinyemi et al. Gender differential in inclination to donate brain for research among Nigerians: the IBADAN Brain Bank Project. Cell Tissue Bank. 2019 Jun;20(2):297-306. doi: 10.1007/s10561-019-09769-4.  We have included the questionnaire as a supplementary material and now heavily summarised the question items in the table displaying some of our results  Due to the nature of the topic, all participants had initial discussions with the data collection team to explain the meaning of certain terms and detailed explanation of the topic. We have included a statement from line 288-294 reading “All participants received a brief education on the concepts being explored in the questionnaire such as informed consent and their meaning and in some cases an explanatory note is provided with the question eg: broad consent (Informed consent only needs to be taken once and this covers for all other use of my sample by researchers) for research on the blood collected from me as it is sometimes practically difficult to re-contact and re-consent participants))”.  The SIREN study has continued with community engagement activities such as outreach and educational programs that provided the platform for recruitment of subjects for this study. As described in the protocol paper of the study (Akinyemi et al, 2020), community - dwelling laypersons were recruited from the seven SIREN participating sites during community engagement programmes regularly organized to promote stroke awareness in the community and also recruit suitable controls for the ongoing study. The number of participants surveyed per site was proportionate to the size of SIREN recruitment from each site. Respondents were selected by stratified random sampling using the list of participants at the community engagement programme list as sampling frame. We have included this statement from line  243-250. | |
| 3 | - Table 1 – Some terms, especially the education levels like “Arabic,” are likely not clear to all readers. It would help if these are defined in the text or as footnote. - In most of the result tables, it is not clear whether the p-values provided indicate significant difference between male and female respondents or between Yes and No responses. At first I thought it was for Yes/No but this doesn’t always make sense. Consider alternative formatting to improve clarity. - Are there regulatory guidelines specific to Africa or from African bodies that you would expect participants to be aware of or was this question asked more generally about any global guideline? - Lines 242-243 – This sentence appears to be missing a data value. - Some of the tables are missing the italicized formatting for survey questions. Also some questions appear to have been only asked of a subset of participants. If so, this should be noted and explained. | We have added a footnote under table 1 to explain the Arabic level of education. It reads as follows  “*Arabic schools are common in Northern Nigeria. They do some forms of formal education, but the focus is Quran. People from Northern Nigeria argue that we cannot equate it to "No formal education*"”.  The P-values represent those for the males and females. The table have been reformatted based on the reviewers’ suggestion to make the p-values clearer.  This question was asked as a general knowledge question and not related to any existing guidelines.  We have not observed this point after reviewing the sentence.  All the tables in the paper have been formatted for placement of p-values and italicising the questions for the participants. | |
|  |  |  |  |
|  | Comments from reviewer 2 |  | |
|  | - From line 154 onwards where the questionnaire is described: this section is hard to read and follow. - My suggestions would be to either provide a paragraph based summary of the questions and include the questionnaire in the supplementary. Any other ways to improve the flow would also be acceptable but I would include a copy of the questionnaire in the supplementary regardless. - Please define all abbreviations used in the text and tables e.g. "Ref" in Table 1 - Please ensure that when quoting a percentage, the presence/lack of space between the number and the % is consistent - Please ensure that the use of decimals (and the number of decimals) is kept consistent throughout the manuscript - With regards to the question "Willingness to donate brain after death", was this question subdivided into just a sample of brain and then another regarding complete brain donation? I suspect that more individuals would have been willing to donate a sample of their brain than their complete brain. - Reformatting of all tables is needed, particularly those reporting questionnaire results; it is difficult to follow as it. All tables should be reviewed for language and grammar errors e.g. Table 3: "I don't just want to" and "Actions for promoting..." - Please rephrase line 247, 89% is not 8 out of 10 respondents - Line 254: 80%" (remove the ") - Inclusion of "however" on line 269 should be removed. - Please rephrase the sentence (and potentially create shorter sentence) from lines 270 till 274. - Table 5: please provide the possible answers (I'm assuming agree/disagree) to the "Perception about informed consent" section - Please rethink how the sentence on line 307 starts with "Age > 50 years..." - Line 357: "concept" should be consent - Please replace all occurence of "just about" with "approximately" - Line 457: Change to "Our study should be considered in light of the following limitations" - Please rephrase how line 464 starts, the inclusion of "established" here is not appropriate. | We thank the reviewer for the comment. We will include the questionnaire in the supplementary material and improve the flow of the section in the paper in a table form to improve the readability.  These have now been defined throughout the paper and the word “Ref” was an error and has been deleted.  This has been edited to make if % without any space between the number and the %.  All values have been rounded to 2 decimal places and p –values to 3 decimal places  The question regarding brain donation is phrased like this in the questionnaire- “ Would you be willing to donate your brain sample for research purposes after death?”  All the tables have been reformatted to improve the consistency, readability and typographical and grammatical errors. The error pointed in Table 3 “I don’t just want to” is not an error but a quote from the questionnaire as it stands.  This has now been rephrased to read, “ Table 3 shows that almost 9 out of 10 (89.0%)”.  Line 254 has been edited to remove (“)  The word “however” is removed from the sentence in line 269.  This sentence has been rephrased and shortened as suggested by the reviewer.  This has been revised to include the percentage agreed for each response.  This has been rephrased to “The odds of sharing of brain tissues with other researchers was 1.8 folds higher among those aged ≥ 50 years as compared to < 50 years”  This has been revised accordingly.  This has been changed as suggested  This line has been edited as such as suggested by the reviewer.  The word established has been changed to “Our study findings demonstrate….” As suggested by the reviewer. | |
|  | **Comments from reviewer 3** |  | |
| 1 | - The article requires a thorough proofread. - There are many grammatical and formatting errors that should be corrected: e.g. Table 7 needs to be reformatted as it is impossible to read the values in the last column; - "sSA" is used as an abbreviation only in the Discussion, and is not defined, even though "sub-Saharan Africa" is used in the Introduction; - the Discussion section needs to be divided into paragraphs. | We thank the reviewer for this comment. We have now thoroughly proofread the manuscript and edited it for grammatical and typographical errors as suggested by the reviewer.  sSA has now been properly defined in the introduction section.  The discussion section has been edited and the paragraphs have been revised. |  |

Many thanks again for the comments, which helped us to improve our manuscript further. We look forward to hearing from you in due course.

On behalf of all the authors.

Yours sincerely

Dr Arti Singh
