# Supplementary material for: Biological sample donation and informed consent for neurobiobanking: Evidence from a community survey in Ghana and Nigeria
Source: PLoS One. 2022 Aug 11;17(8):e0267705. doi: 10.1371/journal.pone.0267705 (PMC9371301; doi:10.1371/journal.pone.0267705)
Supplement: Attachment — Submitted filename: Response to reviewers .docx [file pone.0267705.s004.docx]

08/04/2022

Dear Sir/Madam,

Please find attached our revised manuscript. All the authors have read and approved the revised version of the manuscript which comprises original, unpublished material that has not been considered for publication elsewhere.

We are grateful to the reviewers for their thoughtful comments. We have rewritten the paper in the light of their input and have responded to each comment below.

Detailed response to Reviewers’ comments:

| **Comments from reviewer 1** | | |  |
| --- | --- | --- | --- |
| 1 | **Introduction:** Inconsistencies between “African sub - region” and “African sub region” | We have revised this and made it consistent through out the paper as “African sub region”. | |
| 2 | **Methods:** Line 172 - “1.Demographics” is italicized as though it is a subheader but none of the other items in the numbered list are.  Lines 183-186 - grammar/punctuation problems  **Results:** The table format is improved but spacing and formatting issues remain, including random spaces, inconsistent capitalization, and “%” not be used in all tables.  Table 5 displays the questions differently from previous tables  Tables 6 & 7 formats are difficult to read without more helpful visual formatting and should have similar format.  Lines 236-238 - grammar/punctuation problems  Line 258 - “blood/bank” should be “blood/brain”  Line 276 - “as” should be “are”  **Discussion** Line 376 - there is a redundant comma  Line 378 - there is a redundant word “levels | We have deleted the italicised word (demographics) and rephrased the sentence as  “1. Information on participant demographics includes age, religion, ethnicity, educational qualification, occupation, marital status, monthly income and living arrangement”.  This line has been rewritten to revise the error due to the punctuation.  All tables have been reformatted to make them consistent and include n (%) in all of them where applicable.  Questions in table 5 have been revised to make it consistent with the other tables.  Table 6 and 7 have both been made clearer and reformatted.    This line has been revised  This sentence has been changed to read “blood/brain” as indicated.  This has been changed as requested.  The redundant comma has been deleted as requested  The redundant word “levels” has been deleted. | |

Many thanks again for the comments, which helped us to improve our manuscript further. We look forward to hearing from you in due course.

On behalf of all the authors.

Yours sincerely

Dr Arti Singh
