# Supplementary material for: Biological sample donation and informed consent for neurobiobanking: Evidence from a community survey in Ghana and Nigeria
Source: PLoS One. 2022 Aug 11;17(8):e0267705. doi: 10.1371/journal.pone.0267705 (PMC9371301; doi:10.1371/journal.pone.0267705)
Supplement: Attachment — Submitted filename: PONE-D-21-25570 reviewer comments.docx [file pone.0267705.s002.docx]

PLOS ONE manuscript PONE-D-21-25570

Title: Biological sample donation and informed consent for neurobiobanking: Evidence from a community survey in Ghana and Nigeria

Reviewer comments to authors:

Thank you for this interesting and important manuscript. This report of awareness and understanding of basic elements pertaining to biobanking among African community participants is an important step toward establishing these necessary research resources in Africa. I applaud and encourage these efforts. This manuscript is generally well-written and pertinent to the scientific community, and I recommend it for publication pending minor revisions. The manuscript would benefit from a careful review for grammatical and punctuation errors.

*Introduction*:

- The Introduction appears to be less well-developed than the Discussion and would benefit from elaboration. Specifically, it would help if the authors defined or described certain terms, such as “communal informed consent,” and provided more background on previous studies that were included as comparators in the Discussion.
- There is very little background information provided to explain the inclusion of certain survey questions or answer options.

*Methods:*

- Line 147 – it is not clear why the stroke heritability results from this previous study are relevant in the sample size estimate.
- Lines 171-176 – How were the answer options for the willingness/unwillingness questions determined? These appear to be unique to African communities and differ from what would typically be used in the U.S. or Europe. Explanation of data that informed these would be helpful for the general reader.
- I appreciate the inclusion of all the survey questions in the manuscript but the current format leads to long, run-on sentences. It may be more useful to portray the survey outline as a table or figure.
- It is not clear whether the participants received any brief education on certain topics to ensure they understood the survey questions, such as for the types of consent. This is implied at one point in the Discussion but this should be made clear in the Methods.
- It would help to have more description of how participants were recruited. The authors state that they were selected during SIREN site community engagement activities but what brought them to be involved in these activities and what did these activities entail? Were these individuals patients, caregivers, other? How could this introduce bias?

*Results:*

- Table 1 – Some terms, especially the education levels like “Arabic,” are likely not clear to all readers. It would help if these are defined in the text or as footnotes.
- In most of the result tables, it is not clear whether the p-values provided indicate significant difference between male and female respondents or between Yes and No responses. At first I thought it was for Yes/No but this doesn’t always make sense. Consider alternative formatting to improve clarity.
- Are there regulatory guidelines specific to Africa or from African bodies that you would expect participants to be aware of or was this question asked more generally about any global guideline?
- Lines 242-243 – This sentence appears to be missing a data value.
- Some of the tables are missing the italicized formatting for survey questions. Also some questions appear to have been only asked of a subset of participants. If so, this should be noted and explained.

*Discussion:*

- Line 358 – “sSA” has not been previously defined in the manuscript.
- Lines 361-363 – The authors mention a previous IBADAN study of similar data but this was not mentioned as relevant background in the Introduction. If this is the main comparative work, it would also help to provide a more descriptive comparison of these studies and comment on how that previous study informed the current study.
